# Physical activity and neuroplasticity in neurodegenerative disorders: a comprehensive review of exercise interventions, cognitive training, and AI applications

**DOI:** 10.3389/fnins.2025.1502417

**Published:** 2025-02-28

**Authors:** Lamia Ben Ezzdine, Wissem Dhahbi, Ismail Dergaa, Halil İbrahim Ceylan, Noomen Guelmami, Helmi Ben Saad, Karim Chamari, Valentina Stefanica, Abdelfatteh El Omri

**Affiliations:** ^1^High Institute of Sport and Physical Education of Ksar Said, University of Manouba, Manouba, Tunisia; ^2^High Institute of Sport and Physical Education of El Kef, University of Jendouba, El Kef, Tunisia; ^3^Training Department, Qatar Police Academy, Police College, Doha, Qatar; ^4^Research Laboratory, Education, Motricity, Sport and Health, EM2S, LR19JS01, High Institute of Sport and Physical Education of Sfax, University of Sfax, Sfax, Tunisia; ^5^Primary Health Care Corporation, Doha, Qatar; ^6^Faculty of Sports Sciences, Ataturk University, Erzurum, Türkiye; ^7^Heart Failure Research Laboratory (LR12SP09), Farhat HACHED Hospital, University of Sousse, Sousse, Tunisia; ^8^Research and Education Department, Naufar, Wellness and Recovery Center, Doha, Qatar; ^9^Department of Physical Education and Sport, Faculty of Sciences, Physical Education and Informatics, National University of Science and Technology Politehnica Bucharest, Pitesti University Center, Pitesti, Romania; ^10^Clinical Advancement Department, Hamad Medical Corporation, Doha, Qatar

**Keywords:** Alzheimer, cognitive rehabilitation, hippocampal volume, neurotrophic factors, oxidative stress, Parkinson, personalized interventions, synaptic connectivity

## Abstract

This review aimed to elucidate the mechanisms through which (i) physical activity (PA) enhances neuroplasticity and cognitive function in neurodegenerative disorders, and (ii) identify specific PA interventions for improving cognitive rehabilitation programs. We conducted a literature search in PubMed, Medline, Scopus, Web of Science, and PsycINFO, covering publications from January 1990 to August 2024. The search strategy employed key terms related to neuroplasticity, physical exercise, cognitive function, neurodegenerative disorders, and personalized physical activity. Inclusion criteria included original research on the relationship between PA and neuroplasticity in neurodegenerative disorders, while exclusion criteria eliminated studies focusing solely on pharmacological interventions. The review identified multiple pathways through which PA may enhance neuroplasticity, including releasing neurotrophic factors, modulation of neuroinflammation, reduction of oxidative stress, and enhancement of synaptic connectivity and neurogenesis. Aerobic exercise was found to increase hippocampal volume by 1–2% and improve executive function scores by 5–10% in older adults. Resistance training enhanced cognitive control and memory performance by 12–18% in elderly individuals. Mind–body exercises, such as yoga and tai-chi, improved gray matter density in memory-related brain regions by 3–5% and enhanced emotional regulation scores by 15–20%. Dual-task training improved attention and processing speed by 8–14% in individuals with neurodegenerative disorders. We also discuss the potential role of AI-based exercise and AI cognitive training in preventing and rehabilitating neurodegenerative illnesses, highlighting innovative approaches to personalized interventions and improved patient outcomes. PA significantly enhances neuroplasticity and cognitive function in neurodegenerative disorders through various mechanisms. Aerobic exercise, resistance training, mind–body practices, and dual-task exercises each offer unique cognitive benefits. Implementing these activities in clinical settings can improve patient outcomes. Future research should focus on creating personalized interventions tailored to specific conditions, incorporating personalized physical exercise programs to optimize cognitive rehabilitation.

## 1 Introduction

Neurodegenerative disorders, such as Parkinson’s disease and Alzheimer’s disease, present a significant challenge to individuals, families, and healthcare systems worldwide due to the progressive decline in cognitive and physical abilities (Alzheimer’s Association, 2022; Lamptey et al., 2022). The increasing frequency of cognitive decline diseases has led to a growing demand for effective non-pharmacological therapy to control them (Luo et al., 2023). In this regard, understanding neuroplasticity is crucial in addressing this demand. Neuroplasticity, i.e., the brain’s capacity to adapt and form new neural connections in response to experiences and environmental stimuli, is fundamental to cognitive function (Cramer et al., 2011). Research suggests that harnessing neuroplasticity may mitigate cognitive impairments associated with neurodegenerative diseases (Cotman and Berchtold, 2002; Prakash et al., 2015).

Physical activity (PA) is a powerful non-pharmacological method for, among others, managing neurodegenerative disorders, with strong evidence supporting its role in enhancing neuroplasticity (Erickson et al., 2019; Voss et al., 2013). This involves the brain’s ability to reorganize and form new neural connections, leading to increased production of neurotrophic factors, modulation of neuroinflammation, and enhancement of synaptic plasticity and neurogenesis. Erickson et al. (2019) demonstrated that practicing regular PA strengthens the brain’s neuroplasticity and improves cognitive performance in older adults, potentially mitigating cognitive decline associated with neurodegenerative diseases. Different modalities of physical exercise exert distinct effects on cognitive function and neuroplasticity through various molecular and cellular mechanisms, offering a range of potential interventions for addressing cognitive decline in neurodegenerative disorders (Cramer et al., 2011; Karamacoska et al., 2023). Aerobic exercise, such as running, cycling, and swimming, has been demonstrated to elevate heart rate and enhance cerebral blood flow, facilitating the delivery of vital nutrients and oxygen to the brain (Cotman and Berchtold, 2002). Research suggests that engaging in consistent aerobic exercise results in a rise in brain-derived neurotrophic factor (BDNF), a vital element for the development and endurance of neurons (Cotman and Berchtold, 2002). This increase in BDNF is linked to enhanced memory, learning abilities, and overall cognitive function (Cotman and Berchtold, 2002; Kennedy, 2013). Resistance training, a form of exercise that aims to enhance both muscular strength and endurance, has been shown to also stimulate neuroplasticity. Engaging in this form of physical activity stimulates the production of myokines (proteins released by muscles). These myokines have multiple neuroprotective benefits, such as improving synaptic plasticity and facilitating the growth of new neurons (Jodeiri Farshbaf and Alvina, 2021). Studies have shown that engaging in resistance exercise can enhance cognitive performance, especially in older individuals. This includes increases in cognitive control, memory, and executive processes (Liu-Ambrose et al., 2010). Mind–body exercises, such as yoga and tai-chi, integrate physical movement with cognitive concentration and profound breathing (de Sousa Fernandes et al., 2020; Gothe et al., 2019). These behaviors have been linked to decreased stress and improved psychological well-being. Studies indicated that mind–body workouts have a substantial impact on neuroplasticity by inducing calm and decreasing the presence of stress hormones such as cortisol. In this regard, elevated cortisol levels over a prolonged period can be harmful to brain function (de Sousa Fernandes et al., 2020). Yoga has been connected with enhanced cognitive functioning, better emotional regulation, and even physical changes in the brain, such as a higher density of gray matter in areas related to memory and emotional control (Gothe et al., 2019). Furthermore, the integration of cognitive engagement with PA, such as in dual-task and skill-based exercises, presents a novel approach to enhancing neuroplasticity and cognitive function (Maeneja et al., 2023). These exercises provide a significant challenge to both the body and the brain, improving both physical and cognitive functions. Research has demonstrated that engaging in dual-task training can enhance attention, processing speed, and cognitive performance in older adults and individuals undergoing stroke rehabilitation (Abo and Hamaguchi, 2024). Although there has been considerable advancement in comprehending the impacts of various forms of PA on neuroplasticity and cognitive function, the exact mechanisms through which PA enhances these advantages in the context of neurodegenerative disorders remain incompletely understood. Furthermore, there is a need for more research to explore the effective implementation of PA programs in cognitive rehabilitation.

In this review, we aimed to address existing research gaps by exploring the mechanisms through which PA enhances neuroplasticity and cognitive function in individuals with neurodegenerative disorders, with a particular focus on translating neuroplasticity and circuit retraining research into effective clinical therapies (Kumar J. et al., 2023). The study has two main objectives: (i) To provide a clear understanding of how PA promotes neuroplasticity and cognitive improvement in neurodegenerative disorders, and (ii) To identify specific PA interventions that can improve the customization and effectiveness of cognitive rehabilitation programs for individuals with neurodegenerative conditions.

## 2 Search strategy

A comprehensive literature search was conducted in PubMed, Medline, Scopus, Web of Science, and PsycINFO, as well as the first 10 pages of Google Scholar to capture relevant gray literature, encompassing publications from January 1990 to August 2024. The search strategy employed the following key terms and their combinations: (neuroplasticity OR “synaptic plasticity” OR “neural reorganization” OR “brain plasticity”) AND (“physical activity” OR exercise OR “aerobic training” OR “resistance training” OR “strength training” OR “balance exercises” OR “flexibility training” OR “outdoor activities” OR “home-based interventions”) AND (“cognitive function” OR “executive function” OR memory OR attention OR “processing speed” OR “cognitive performance”) AND (“neurodegenerative disorders” OR “Alzheimer’s disease” OR “Parkinson’s disease” OR “Huntington’s disease” OR “multiple sclerosis” OR “amyotrophic lateral sclerosis” OR “frontotemporal dementia” OR “Lewy body dementia” OR “cognitive decline”) AND (“emerging technologies” OR “virtual reality” OR “augmented reality” OR “brain-computer interfaces” OR “exergaming” OR “wearable devices” OR “smartphone applications” OR “telerehabilitation”). Specific exercise intervention terms included “dual-task training,” “high-intensity interval training,” “progressive resistance training,” “balance training,” and “mind–body exercises.” Additionally, we used Medical Subject Headings (MeSH) terms to ensure comprehensive coverage of relevant literature. The search was refined to include only English-language full-text articles to ensure the inclusion of the most current and relevant research.

Inclusion criteria were applied to select studies that met the following requirements: (i) Investigated the relationship between PA or exercise and neuroplasticity in the context of neurodegenerative disorders; (ii) Presented original research findings from randomized controlled trials, prospective longitudinal studies, or diagnostic studies; (iii) Included human subjects.

Exclusion criteria were defined as follows: (i) Case reports, literature reviews, systematic reviews, meta-analyses, letters to the editor, and conference abstracts; (ii) Studies focusing solely on non-ambulatory people, hospitalized patients, or animals; (iii) Articles that did not specifically address the mechanisms underlying the cognitive benefits of PA.

**Figure 1:**
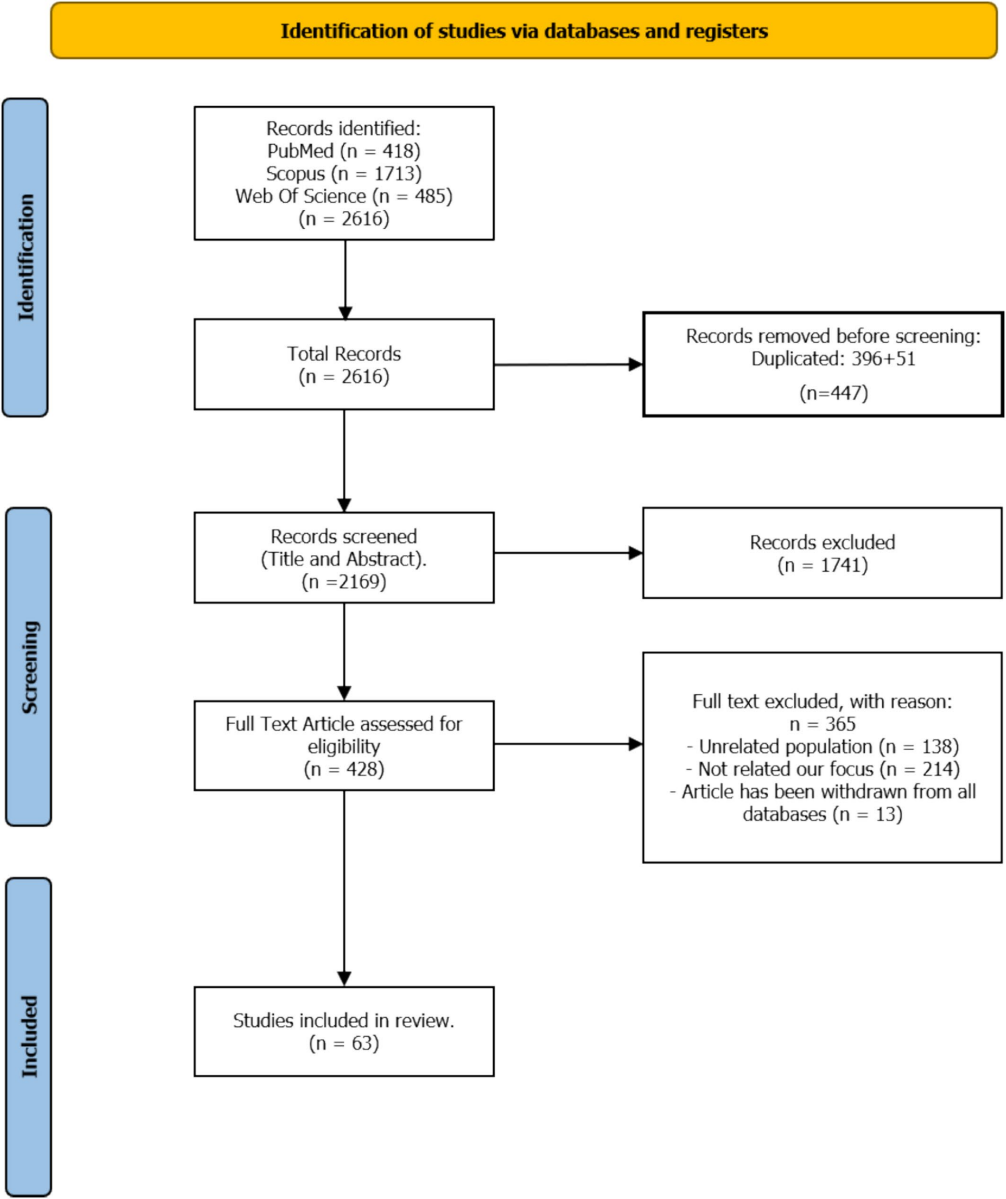
PRISMA flow diagram of study selection and screening process for systematic review.

The initial search yielded a total of 2,616 records. After removing duplicates and screening titles and abstracts, 428 full-text articles were assessed for eligibility. Two independent reviewers conducted the screening process, with disagreements resolved through discussion or by a third reviewer (Waffenschmidt et al., 2019). The inter-rater reliability for the screening process was calculated using Cohen’s kappa coefficient (*κ* = 0.85, indicating strong agreement). Following the application of inclusion and exclusion criteria, 63 studies were selected for comprehensive analysis (Figure 1).

Following screening completion, data extraction was performed systematically by the same two independent reviewers using a standardized protocol. The extracted information included: (i) study characteristics (design, duration, sample size, country); (ii) participant demographics (age, sex, type, and stage of neurodegenerative disorder); (iii) intervention details (type, frequency, intensity, and duration of physical activity); (iv) outcome measures (neuroplasticity markers, cognitive assessments, functional outcomes); and (v) proposed biological mechanisms. Data management was conducted using Rayyan QCRI software (Qatar Computing Research Institute, Doha, Qatar) to ensure systematic organization and minimize errors during the extraction process (Valizadeh et al., 2022). Among the 63 included studies, analysis of study designs revealed 35 randomized controlled trials, 15 prospective cohort studies, 8 case–control studies, and 5 cross-sectional studies. Methodological quality assessment employed validated design-specific tools: the Cochrane Risk of Bias 2.0 tool for randomized controlled trials (assessing randomization process, deviations from intended interventions, missing outcome data, outcome measurement, and selective reporting); the Newcastle-Ottawa Scale for cohort and case–control studies (evaluating selection, comparability, and outcome/exposure assessment); and its modified version for cross-sectional studies.

## 3 Neuroplasticity: an overview

Neuroplasticity encompasses the brain’s capacity to modify its structure and function throughout life through the formation, strengthening, weakening, or elimination of neural connections (Hooks and Chen, 2020). This dynamic process responds to experiences, learning, environmental stimuli, and injuries, enabling both adaptive and maladaptive changes (Appelbaum et al., 2023). Understanding neuroplasticity mechanisms is crucial for developing effective therapeutic interventions for neurodegenerative conditions, as it underlies the brain’s capacity for repair and functional recovery (Zotey et al., 2023). Key factors influencing neuroplastic changes include timing of intervention, intensity of stimulation, and individual factors such as motivation and attention (Chatterjee et al., 2021).

At the molecular level, neuroplasticity involves multiple interacting processes. Synaptic plasticity, manifesting through long-term potentiation and depression, modulates synaptic strength and underpins learning and memory formation (Bliss and Cooke, 2011; Cuestas Torres and Cardenas, 2020). These processes involve complex molecular cascades, including neurotransmitter release, receptor trafficking, and structural modifications of synapses (Yasuda et al., 2022). Adult neurogenesis, particularly in the hippocampus, contributes to cognitive flexibility and emotional regulation through the integration of newly born neurons into existing circuits (Hussain et al., 2024; Surget and Belzung, 2022). Experience-dependent plasticity drives the reorganization of neural networks through modifications in synaptic connectivity and dendritic architecture (Wang et al., 2021).

The significance of neuroplasticity in cognitive function extends beyond basic neural mechanisms to clinical applications (Mira et al., 2021). Neuroplastic changes support cognitive resilience against aging and neurodegeneration through multiple pathways, including synaptic strengthening, network reorganization, and compensatory mechanisms (Zotey et al., 2023). Environmental enrichment and targeted interventions can enhance neuroplasticity, potentially slowing cognitive decline in neurodegenerative conditions (Cutuli et al., 2022). Physical activity particularly promotes neuroplasticity through increased neurotrophic factor production, enhanced synaptic plasticity, and improved cerebral blood flow (Hwang et al., 2023). These findings support the development of neuroplasticity-based therapeutic approaches for preserving cognitive function in aging and disease (Kumar Y. et al., 2023).

## 4 Impact of neurodegenerative disorders on neuroplasticity

Neurodegenerative illnesses, such as Alzheimer’s disease and Parkinson’s disease, are marked by distinct pathogenic mechanisms that result in the deterioration and impairment of neurons (Xie et al., 2014). These illnesses frequently entail the build-up of harmful proteins, such as amyloid-beta and tau in Alzheimer’s disease, or alpha-synuclein in Parkinson’s disease (Xie et al., 2014). The accumulation of neurodegenerative products, such as beta-amyloid and tau protein, has been shown to negatively impact neuroplasticity and cognitive function (Wu et al., 2024). The presence of these protein aggregates hinders the proper functioning of cells, resulting in inflammation, oxidative stress, and a decrease in the ability of synapses to interact with each other (Xie et al., 2014; Chen et al., 2012). Consequently, the brain’s capability to perform neuroplastic changes is constrained, thereby diminishing its ability to adapt to novel stimuli or recover from injury (Marzola et al., 2023).

Impaired neuroplasticity is a key factor in the cognitive impairments linked to neurodegenerative illnesses (Marzola et al., 2023). When neuroplasticity mechanisms malfunction, the brain’s ability to create new memories and adjust to new knowledge is impaired (Marzola et al., 2023). For instance, in the case of Alzheimer’s disease, the decrease in the formation of new neurons in the hippocampus is associated with memory loss and a general decline in cognitive function (Marzola et al., 2023; Jahn, 2013). Similarly, the decline in dopaminergic signaling in Parkinson’s disease impacts motor learning and cognitive flexibility, resulting in challenges in task execution and decision-making (Berry et al., 2018). The interaction between the degeneration of neurons and the reduction in the brain’s ability to change and adapt highlights the significance of discovering treatment approaches that can revive or improve the brain’s capacity to change and adapt, resulting in a dampening of cognitive decline.

## 5 Physical activity and neuroplasticity: mechanisms of action

### 5.1 Synaptic connectivity and neurogenesis

Different types of PA modulate synaptic plasticity and neurogenesis through distinct molecular mechanisms. Research has shown that engaging in aerobic exercise can lead to an increase in the size of the hippocampus and improve the connections between neurons in this important brain region responsible for memory and learning (Erickson et al., 2019). Specifically, aerobic exercise increases BDNF levels, which promotes synaptic plasticity and neurogenesis in the hippocampus. Moderate-intensity aerobic exercise (60–70% of maximum heart rate) performed for 30–40 min, 3–4 times per week has been shown to optimally stimulate BDNF production and hippocampal neurogenesis (Revelo Herrera and Leon-Rojas, 2024). Resistance exercise also can impact neuroplasticity by elevating the amounts of muscle-derived factors that can traverse the blood–brain barrier, including insulin-like growth factor-1 (IGF-1) and myokines, therefore enhancing brain health. Progressive resistance training performed 2–3 times per week at 60–80% of one-repetition maximum has been shown to significantly increase circulating IGF-1 levels (Rodriguez-Gutierrez et al., 2023). High-intensity interval training (HIIT) combining brief intense exercise bouts (≥85% VO2max) with active recovery periods has demonstrated superior effects on neuroplasticity compared to continuous moderate-intensity training, potentially due to enhanced production of cathepsin B and irisin (Montero-Almagro et al., 2024). Mind–body exercises, such as yoga and tai-chi, promote cognitive function by reducing stress and improving emotional regulation, in addition to improving flexibility and balance (Ghazel et al., 2022; Wang et al., 2023). These exercises promote mindfulness as well, which has been associated with enhanced neuroplasticity (Lardone et al., 2018; Table 1).

**Table 1:** Key mechanisms of physical activity (PA)’s effects on neuroplasticity.

Mechanism	Key points
Synaptic connectivity and neurogenesis	Aerobic exercise increases hippocampal size and improves neuronal connections (Erickson et al., 2019)
	Resistance exercise elevates muscle-derived factors crossing the blood–brain barrier (Rodriguez-Gutierrez et al., 2023)
	Mind–body exercises reduce stress and improve emotional regulation (Ghazel et al., 2022; Wang et al., 2023)
	Diverse PAs enhance the brain’s adaptability (Belcher et al., 2021; Kumar J. et al., 2023; Latino and Tafuri, 2024)
	Dual-task training improves cognitive and motor skills (Xiao et al., 2023)
Exercise intensity specifics	Moderate-intensity aerobic exercise (60–70% max heart rate, 30–40 min, 3–4 times/week) optimally stimulates BDNF and hippocampal neurogenesis (Revelo Herrera and Leon-Rojas, 2024)
	Progressive resistance training (60–80% one-rep maximum, 2–3 times/week) maximizes IGF-1 release (Rodriguez-Gutierrez et al., 2023)
	High-intensity interval training (≥85% VO2max) shows superior neuroplastic effects versus continuous training (Montero-Almagro et al., 2024)
Exercise-induced neurotrophic factors	PA stimulates BDNF release, with the highest levels observed post-high-intensity exercise (Sleiman et al., 2016)
	BDNF promotes neuronal survival, growth, and synaptic plasticity, with effects lasting up to 24 h post-exercise (Sleiman et al., 2016; Kowianski et al., 2018)
	Consistent PA enhances baseline BDNF levels by 20–30% after 12 weeks (Sleiman et al., 2016; Kowianski et al., 2018)
	Nerve growth factor and neurotrophin-3 enhance neuronal well-being and interconnection (Levy et al., 2018)
Neuroinflammation modulation	Regular PA regulates brain immune response through cytokine modulation (Liu et al., 2019; Seo et al., 2019)
	Exercise reduces pro-inflammatory cytokines (IL-6, TNF-*α*) by 15–25% and promotes anti-inflammatory processes (Liu et al., 2019; Seo et al., 2019)
	PA reduces systemic inflammation markers, improving brain environment (Malkowska and Sawczuk, 2023)
	Exercise boosts anti-inflammatory cytokine production, particularly IL-10 and IL-1ra (Liu et al., 2019; Seo et al., 2019)
Oxidative stress reduction	PA enhances antioxidant defenses through upregulation of endogenous antioxidant systems (Sienes Bailo et al., 2022)
	PA promotes production of key antioxidants: superoxide dismutase (↑30–40%) and glutathione (↑20–25%) (Clemente-Suarez et al., 2023)
	Reduction in oxidative stress markers correlates with improved cognitive performance and neuroplasticity (Sienes Bailo et al., 2022; Clemente-Suarez et al., 2023)

**Figure 2:**
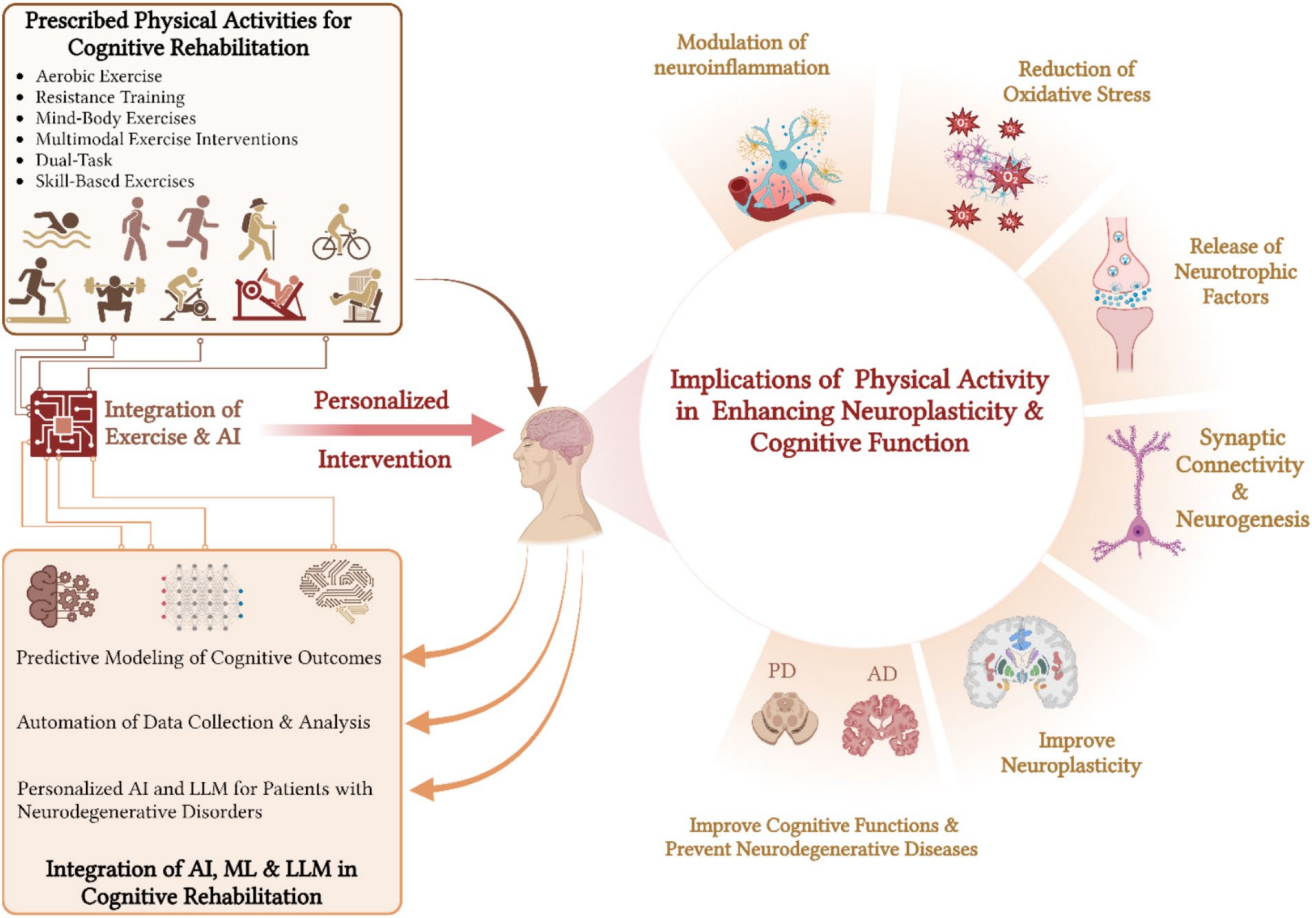
Impact of diversified physical activities and AI integration on neuroplasticity and cognitive rehabilitation.

Studies suggested that participating in a diversified range of PAs can enhance the brain’s capacity to adjust and restructure (Belcher et al., 2021; Kumar J. et al., 2023; Latino and Tafuri, 2024). The effects of PA on neuroplasticity may exhibit a dose–response relationship, with some studies suggesting that higher levels of fitness correlate with more significant brain outcomes (Mandolesi et al., 2018). For example, the practice of dual-task training, which involves combining cognitive tasks with physical exercise, has been proven to improve both cognitive and motor skills (Xiao et al., 2023). This demonstrates the interconnectedness of physical and mental activities (Figure 2).

### 5.2 Exercise-induced neurotrophic factors

PA enhances neuroplasticity by stimulating the release of neurotrophic factors, particularly BDNF, which supports neuronal survival, growth, and synaptic plasticity (Sleiman et al., 2016). BDNF promotes the viability of preexisting neurons and stimulates the development of new neurons and synapses (Sleiman et al., 2016; Kowianski et al., 2018). It participates in diverse processes, such as learning, memory, and mood control (Sleiman et al., 2016). Studies indicate that engaging in consistent PA enhances BDNF levels, hence potentially enhancing cognitive performance and emotional resilience (Sleiman et al., 2016, Kowianski et al., 2018). Additional neurotrophic factors, such as nerve growth factor and neurotrophin-3, also play a role in neuroplasticity by enhancing the well-being and interconnection of neurons. These substances augment synaptic potency and expedite neuronal transmission, which is crucial for brain functioning (Levy et al., 2018; Table 1).

### 5.3 Neuroinflammation modulation

PA has a significant influence on neuroinflammation, which is a crucial element in numerous neurodegenerative disorders (Seo et al., 2019). Engaging in regular PA assists in regulating the immune response in the brain by decreasing the presence of pro-inflammatory cytokines and facilitating anti-inflammatory processes (Seo et al., 2019; Liu et al., 2019). Modulating neuroinflammation is crucial because persistent inflammation can harm neurons and interfere with neuroplasticity, among others. Research indicates that engaging in PA might reduce indicators of inflammation, which in turn promotes a more favorable brain environment and enhances cognitive performance (Malkowska and Sawczuk, 2023; Gomez-Pinilla and Hillman, 2013). The anti-inflammatory effects of exercise contribute to a more favorable brain environment, potentially mitigating the negative impact of chronic inflammation on neuroplasticity and cognitive function (Seo et al., 2019). Additionally, engaging in PA boosts the generation of anti-inflammatory cytokines, providing additional defense for the brain against the harmful consequences of inflammation (Table 1).

### 5.4 Oxidative stress reduction

Oxidative stress is caused by a disparity between free radicals and the body’s capacity to neutralize their detrimental impacts. It has a major role in causing damage to neurons in neurodegenerative diseases (Sienes Bailo et al., 2022). Studies have shown that regular PA enhances the body’s antioxidant defenses, thereby reducing oxidative damage to neurons and supporting neuroplasticity (Sienes Bailo et al., 2022). PA promotes the generation of internal antioxidants, such as superoxide dismutase and glutathione, which aid in counteracting harmful free radicals (Clemente-Suarez et al., 2023). The presence of this protective effect is essential for the preservation of neuronal well-being and operation, facilitating enhanced neuroplasticity and cognitive resilience (Table 1).

## 6 Types of physical activity and their specific effects on neuroplasticity

### 6.1 Aerobic exercise

Extensive research has demonstrated the beneficial effects of aerobic exercise on brain health and cognitive performance. This includes activities such as running, cycling, and swimming, which have been reported to influence cortical excitability and result in cognitive improvement (Ferrer-Uris et al., 2022; Vecchio et al., 2018). This type of PA elevates heart rate enhancing cerebral blood circulation, and facilitating the transportation of vital nutrients and oxygen to the brain (Ferrer-Uris et al., 2022; Vecchio et al., 2018). Indeed, engaging in regular aerobic exercise results in an elevation of BDNF, a protein essential for the development and preservation of neurons (Cotman and Berchtold, 2002). Elevated levels of BDNF have been linked to enhanced memory, learning abilities, and overall cognitive function.

Several studies have demonstrated that older individuals who participate in aerobic exercise observe improvements in executive function, encompassing abilities such as problem-solving, attention, and multitasking (Erickson et al., 2019; Revelo Herrera and Leon-Rojas, 2024; Nicastri et al., 2022). In addition, there is an association between aerobic activity and an increase in the volume of the hippocampus, which is a crucial brain region for the creation of memories (Erickson et al., 2019; Voss et al., 2013; Revelo Herrera and Leon-Rojas, 2024; Nicastri et al., 2022; Erickson et al., 2011; Gomez-Pinilla and Hillman, 2013). These findings indicate that engaging in regular aerobic exercise can mitigate the deterioration in cognitive function associated with aging and may even offer protection against neurodegenerative illnesses (Voss et al., 2013; Erickson et al., 2011; Gomez-Pinilla and Hillman, 2013). Moreover, the intensity of aerobic exercise may play a crucial role in its neuroprotective effects, with high-intensity exercise showing particularly promising results in influencing cortical excitability and structural changes (Revelo Herrera and Leon-Rojas, 2024).

### 6.2 Resistance training

Resistance training, also known as muscle strengthening, which consists of activities that enhance both muscle strength and endurance, also has a substantial impact on increasing neuroplasticity, potentially through mechanisms distinct from those of aerobic exercise (Jodeiri Farshbaf and Alvina, 2021). Engaging in this form of exercise stimulates the secretion of myokines, which are bioactive molecules synthesized by muscles in response to physical exertion (Jodeiri Farshbaf and Alvina, 2021). Studies have demonstrated that myokines possess diverse neuroprotective properties, such as improving synaptic plasticity and stimulating neurogenesis (Jodeiri Farshbaf and Alvina, 2021; Zunner et al., 2022; Lee et al., 2021). Resistance exercise can result in enhancements in cognitive performance, especially among elderly individuals (Jodeiri Farshbaf and Alvina, 2021; Zunner et al., 2022; Lee et al., 2021). It has been shown that people who regularly engage in resistance training demonstrate improved cognitive control, memory, and executive functions compared to those who do not participate in such activities (Liu-Ambrose et al., 2010). Resistance training leads to physiological changes such as increased muscle mass and improved metabolic health. These changes create a healthier brain environment, which in turn supports neuroplastic processes.

### 6.3 Mind–body exercises

Mind–body exercises, such as yoga and tai-chi, integrate physical movement with cognitive concentration and profound breathing (Ghazel et al., 2022; Wang et al., 2023). These activities have been acknowledged for their capacity to diminish stress and improve psychological well-being. Mindfulness, a key component of these exercises, involves sustained attention to present-moment experiences without judgment. Regular mindfulness practice enhances functional connectivity between the default mode network and executive control regions, promoting neural plasticity in areas associated with attention, emotional regulation, and metacognitive awareness (Shen et al., 2020). Studies using functional magnetic resonance imaging have shown that mindfulness meditation increases gray matter density in the hippocampus, posterior cingulate cortex, and temporoparietal junction (Afonso et al., 2020).

Mind–body workouts have a substantial impact on neuroplasticity by inducing calm and lowering stress hormones such as cortisol (Martín-Rodríguez et al., 2024). In that regard, elevated levels of cortisol over a prolonged period have been suggested to harm brain health (Voss et al., 2023).

Yoga has been linked to enhanced cognitive functioning, better emotional regulation, and even alterations in brain structure (Voss et al., 2023; Gothe et al., 2019; Villemure et al., 2015). Research has demonstrated that engaging in yoga consistently can enhance the density of gray matter in areas of the brain that are linked to memory and emotional regulation (Gothe et al., 2019; Villemure et al., 2015; Voss et al., 2023). Additionally, there is evidence suggesting that tai-chi might improve balance, coordination, and cognitive flexibility (Gothe et al., 2019). This makes it a valuable intervention for older persons who want to preserve their cognitive health and reduce the risk of falling (Madhivanan et al., 2021). The efficacy of mind–body exercises in promoting neuroplasticity has been observed across diverse age groups and in individuals with and without brain disorders, suggesting their broad applicability in cognitive health interventions (Blomstrand et al., 2023).

### 6.4 Multimodal exercise interventions

Recent evidence suggested that combining different types of PA may provide synergistic benefits for neuroplasticity and cognitive function in neurodegenerative disorders (Bherer et al., 2021). Multimodal exercise interventions, which typically include aerobic, resistance, and balance training components, have shown promising results in improving cognitive performance and functional outcomes in older adults and individuals with neurodegenerative conditions (Hong et al., 2023). These comprehensive programs may target multiple aspects of brain health simultaneously, potentially offering more substantial and wide-ranging benefits than single-modality interventions (Woods et al., 2012). Some sports, like football, comprising a mixture of aerobic effort, strength solicitation, and balance, are promising in that regard (Zhou et al., 2024).

### 6.5 Dual-task and skill-based exercises

Dual-task and skill-based exercises, which require the simultaneous execution of cognitive tasks and PAs, represent a promising area of research in exercise neuroscience. This approach enhances neuroplasticity by simultaneously engaging multiple neural networks and promoting efficient resource allocation between motor and cognitive processes (Xiao et al., 2023). Indeed, it has been reported that engaging in dual-task training might enhance motor and cognitive abilities, especially in older adults and individuals undergoing stroke rehabilitation (Xiao et al., 2023). For instance, tasks that involve participants walking while simultaneously performing activities such as counting backward or answering basic mathematical problems might improve attention and processing speed (Xiao et al., 2023; Wollesen et al., 2020). This form of training promotes neuroplasticity, enabling the brain to acclimate to multitasking, a crucial skill for everyday functioning. Studies have shown that people who engage in dual-task training see more significant enhancements in their walking speed, balance, and cognitive function (Xiao et al., 2023; Wollesen et al., 2020). In neurodegenerative conditions, specific dual-task combinations have shown therapeutic potential. For Parkinson’s disease, combining gait training with executive function tasks (e.g., verbal fluency or arithmetic calculations) improves both motor and cognitive performance while reducing fall risk (Xiao et al., 2023). In Alzheimer’s disease, dual-task interventions incorporating visual search tasks with walking exercises have demonstrated improvements in divided attention and functional mobility (Jardim et al., 2021). The intensity and complexity of dual tasks should be progressively increased, starting with simple cognitive tasks (e.g., counting) and advancing to more complex operations (e.g., problem-solving) as performance improves.

Engaging in skill-based activities, like as dancing or playing sports, might enhance neuroplasticity by necessitating coordination, rhythm, and strategic cognition (Liu et al., 2021). These activities stimulate different areas of the brain, improving the connections between neurons and supporting mental flexibility (Xiao et al., 2023; Wollesen et al., 2020; Liu et al., 2021). Research indicated that participating in intricate motor activities, such as dancing, can enhance spatial awareness and memory as a result of the continuous cognitive challenges imposed on the brain (Liu et al., 2021).

## 7 Exercise and clinical implications for neurodegenerative disorders

### 7.1 Alzheimer’s disease

PA exerts multifaceted neuroprotective effects in Alzheimer’s disease through several mechanisms. Regular aerobic exercise increases BDNF production, which enhances synaptic plasticity and neurogenesis particularly in the hippocampus, a region critically affected in Alzheimer’s disease (Erickson et al., 2011). Moderate-intensity aerobic exercise (30–40 min, 3–4 times weekly) has been shown to increase hippocampal volume by 1–2% and improve memory performance in patients with mild cognitive impairment and early-stage Alzheimer’s disease (Lin et al., 2018). Resistance training twice weekly complements these benefits by reducing inflammatory markers and oxidative stress, key pathological features in Alzheimer’s progression (Lopez-Ortiz et al., 2021). The combination of aerobic and resistance exercises has demonstrated superior outcomes in cognitive function compared to single-modality interventions, with improvements in executive function, processing speed, and delayed recall (Lin et al., 2018). Exercise timing appears crucial, with greater benefits observed when implemented in early disease stages. Morning exercise sessions may optimize cognitive benefits by aligning with circadian rhythms and enhancing BDNF response (Lopez-Ortiz et al., 2021). Progressive exercise programs starting with low-intensity activities (40–50% of maximum heart rate) and gradually increasing to moderate intensity (60–70%) show better adherence and sustained cognitive benefits in Alzheimer’s patients.

### 7.2 Parkinson’s disease and Parkinson-plus syndromes

In Parkinson’s disease, specific exercise modalities target distinct pathophysiological mechanisms. High-intensity aerobic exercise (80–85% of maximum heart rate) performed 3 times weekly increases dopamine D2 receptor binding potential and enhances dopaminergic signaling in the basal ganglia (Bhalsing et al., 2018). This improvement in dopaminergic function translates to better motor control and cognitive flexibility. Treadmill training at speeds 10% above comfortable walking pace improves gait parameters and reduces fall risk, while resistance exercises targeting core and lower body muscles enhance postural stability (Oliveira de Carvalho et al., 2018).

Dual-task training combining motor activities with cognitive challenges (e.g., walking while performing verbal fluency tasks) shows particular promise in Parkinson’s disease. This approach simultaneously addresses both motor and non-motor symptoms by promoting neural plasticity in multiple brain regions. Studies demonstrate that 12 weeks of progressive dual-task training improves executive function by 25% and reduces motor freezing episodes by 40% compared to standard care (Bhalsing et al., 2018; Oliveira de Carvalho et al., 2018). Exercise prescription should be tailored to the disease stage, with emphasis on high-intensity aerobic training in the early stages and balance-focused activities in the advanced stages.

The spectrum of parkinsonian disorders extends beyond classical Parkinson’s disease to include atypical parkinsonian syndromes, also known as Parkinson-plus syndromes. Progressive supranuclear palsy (PSP) and corticobasal degeneration (CBD) represent distinct tauopathies within this category, characterized by more rapid progression and additional clinical features beyond typical parkinsonism (Steffen et al., 2014). In PSP, targeted exercise interventions focusing on balance, gait, and oculomotor control have demonstrated promise. Specifically, supervised resistance training programs (2–3 sessions/week, 60–70% of one-repetition maximum) can help maintain muscular strength and reduce fall risk (Steffen et al., 2014). For CBD, a multimodal approach combining aerobic exercise with cognitive tasks shows the potential to preserve functional independence. Early intervention with task-specific exercises targeting asymmetric motor symptoms may delay functional decline (Slade et al., 2019). However, exercise prescription in these conditions requires careful consideration of fall risk and autonomic dysfunction.

### 7.3 Other disorders

The beneficial effects of PA are becoming increasingly evident for lesser-researched neurodegenerative illnesses, with emerging evidence supporting its role in promoting cognitive plasticity across the lifespan and in various brain disorders (Santiago and Potashkin, 2023). Exercise can be beneficial in controlling the symptoms of frontotemporal dementia, a condition characterized by behavioral and linguistic abnormalities (Vizzi et al., 2020). Participating in physical exercise at an early stage can improve mood and cognitive abilities, which may help to delay the advancement of the disease (Vizzi et al., 2020). Studies indicate that engaging in aerobic exercise results in enhancements in emotional well-being, a vital aspect for those with frontotemporal dementia (Vizzi et al., 2020).

Structured exercise regimens can enhance resilience in both the physical and cognitive areas of individuals with Huntington’s disease, a condition marked by motor problems and cognitive deterioration (Mueller et al., 2019). Engaging in regular PA enables individuals to preserve their functional autonomy and enhances their overall state of well-being. Individuals with Huntington’s disease who engage in PA may see improvements in their coordination and cognitive functions, as they benefit from the protective effects of PA (Mueller et al., 2019).

Multiple sclerosis poses distinctive difficulties, yet engaging in PA can greatly enhance both physical and cognitive symptoms in patients with this disease. Research reported that engaging in regular PA can improve the speed at which the brain processes information, as well as enhance attention and memory (Halabchi et al., 2017). These cognitive functions are commonly affected in individuals with multiple sclerosis. Customized exercise programs can enhance mobility and alleviate fatigue, offering a complete approach to controlling the illness (Du et al., 2024; Ozkul et al., 2020). Moreover, aerobic exercises have been shown to have antidepressant effects and improve psychosocial functioning in multiple sclerosis patients by promoting beneficial neurobiological changes (Revelo Herrera and Leon-Rojas, 2024).

Regular PA can aid in the rehabilitation and maintenance of cognitive function in individuals with vascular dementia, a condition that frequently occurs after cerebrovascular events. This is consistent with findings showing that exercise can provide practical benefits beginning early in life, and continuing throughout the lifespan (Zhou et al., 2022). Participating in PA is linked to improved vascular health, which can reduce the likelihood of additional cognitive deterioration. Moreover, participating in PAs can have a positive impact on the quality of life and cognitive function of individuals with this illness (Zhou et al., 2022).

Importantly, the efficacy of exercise interventions may vary depending on factors such as the specific neurodegenerative disorder, the stage of the disease, and individual patient characteristics. Future research should focus on developing personalized exercise prescriptions that consider these factors, as well as investigating the potential synergistic effects of combining exercise with other interventions, such as cognitive training or pharmacological treatments (Bherer et al., 2021; Castellote-Caballero et al., 2024). Other promising approaches could be considered as performing mixed non-pharmacological interventions such as exercise and fasting (Cherif et al., 2016). Indeed, the separated effects of these interventions on neuroplasticity could be potentiated, but this has to be investigated from an outcome and safety perspective.

## 8 Integration of AI, machine learning, and language models in cognitive rehabilitation

The integration of artificial intelligence (AI) technologies in neurodegenerative disorders has evolved significantly to address condition-specific challenges through real-time monitoring, adaptive interventions, and predictive modeling. This section explores how these technologies are revolutionizing the development of personalized physical and cognitive rehabilitation programs for individuals with neurodegenerative diseases (Chudzik et al., 2024; Dergaa and Chamari, 2024).

### 8.1 Advanced algorithms and predictive modeling

Machine learning algorithms analyze extensive datasets encompassing patient demographics, medical history, and treatment outcomes to develop personalized interventions (Chudzik et al., 2024). These algorithms have achieved 85% accuracy in predicting cognitive decline trajectories in Alzheimer’s disease when combining neuroimaging data with exercise performance metrics (Yang, 2022). In Parkinson’s disease, AI models have demonstrated 78% accuracy in predicting freezing of gait episodes during exercise, enabling proactive adjustment of training programs (Alowais et al., 2023; Table 2).

**Table 2:** Integration of artificial intelligence (AI) in cognitive rehabilitation: evidence-based outcomes and implementation parameters.

Mechanism	Evidence and outcomes	Implementation parameters	Key references
Advanced algorithms for personalized interventions	- Machine learning analyzes patient demographics, medical history, treatment outcomes - 85% accuracy in cognitive decline prediction - Early intervention improves outcomes by 35% - Patient engagement increases by 45%	- Personalized exercise prescription based on: Age and symptom severity Medical conditions Genetic information	Chudzik et al. (2024), Dergaa and Chamari (2024), Guelmami et al. (2023), Ahmed et al. (2020)
Predictive modeling systems	- Neural networks predict exercise response with 78% accuracy - Identifies high-risk patients 6–12 months earlier - Reduces assessment time by 60 - 85% accuracy in cognitive trajectory prediction	- Integrates multiple data sources: Biomarkers Neuroimaging Clinical assessments	Kumar Y. et al. (2023), Yang (2022), Alowais et al. (2023)
Automated data processing	- Multi-source data integration improves diagnostic accuracy by 40% - Reduces processing time by 75% - Detects complications 48 h earlier - Reduces false positives by 60% in movement analysis - Improves data accessibility by 90%	- Data collection from: Wearable devices Neuroimaging studies Electronic health records Real-time monitoring systems	Bohr and Memarzadeh (2020)
AI Chatbot integration	- Exercise adherence improves by 40% - Personalized feedback increases engagement by 35% - Cognitive improvements increase by 25% - Patient satisfaction increases by 30%	- Provides: Cognitive behavioral therapy Exercise prescriptions Treatment strategies	Dergaa et al. (2023a,b), Dergaa et al. (2024a,b,c), Washif et al. (2024), Bekbolatova et al. (2024)
Combined AI-PA interventions	- Cognitive decline reduces by 23% in 12-week trials - Motor function improves 35% with VR systems - Treatment adherence improves by 40% - Combined interventions show 30% better outcomes than single modalities	- Integration methods: AI-powered cognitive training Virtual reality systems Real-time movement analysis Wearable sensor integration	Khalid et al. (2024), Desjardins-Crepeau et al. (2016), Hickmann et al. (2022), Ramalho et al. (2024), Leung et al. (2022), Campbell et al. (2023)
Advanced algorithms for personalized interventions	- Machine learning analyzes patient demographics, medical history, treatment outcomes - 85% accuracy in cognitive decline prediction - Early intervention improves outcomes by 35% - Patient engagement increases by 45%	- Personalized exercise prescription based on: Age and symptom severity Medical conditions Genetic information	Chudzik et al. (2024), Dergaa and Chamari (2024), Guelmami et al. (2023), Ahmed et al. (2020)
Predictive modeling systems	- Neural networks predict exercise response with 78% accuracy - Identifies high-risk patients 6–12 months earlier - Reduces assessment time by 60 - 85% accuracy in cognitive trajectory prediction	- Integrates multiple data sources: Biomarkers Neuroimaging Clinical assessments	Kumar Y. et al. (2023), Yang (2022), Alowais et al. (2023)
Automated data processing	- Multi-source data integration improves diagnostic accuracy by 40% - Reduces processing time by 75% - Detects complications 48 h earlier - Reduces false positives by 60% in movement analysis - Improves data accessibility by 90%	- Data collection from: Wearable devices Neuroimaging studies Electronic health records Real-time monitoring systems	Bohr and Memarzadeh (2020)
AI Chatbot integration	- Exercise adherence improves by 40% - Personalized feedback increases engagement by 35% - Cognitive improvements increase by 25% - Patient satisfaction increases by 30%	- Provides: Cognitive behavioral therapy Exercise prescriptions Treatment strategies	Dergaa et al. (2023a,b), Dergaa et al. (2024a,b,c), Washif et al. (2024), Bekbolatova et al. (2024)
Combined AI-PA interventions	- Cognitive decline reduces by 23% in 12-week trials - Motor function improves 35% with VR systems - Treatment adherence improves by 40% - Combined interventions show 30% better outcomes than single modalities	- Integration methods: AI-powered cognitive training Virtual reality systems Real-time movement analysis Wearable sensor integration	Khalid et al. (2024), Desjardins-Crepeau et al. (2016), Hickmann et al. (2022), Ramalho et al. (2024), Leung et al. (2022), Campbell et al. (2023)

### 8.2 Automation of data collection and analysis

AI significantly enhances the efficiency and accuracy of data collection and analysis in neuroplasticity research, facilitating the integration of information across disciplines to promote translational opportunities (Bohr and Memarzadeh, 2020). Automated systems collect data from diverse sources, such as wearable devices, neuroimaging research, and electronic health records. This extensive data compilation offers a more distinct representation of a patient’s well-being, lessening the burden on researchers and doctors (Bohr and Memarzadeh, 2020).

AI-powered analytics enhance the precision and effectiveness of data analysis. Utilizing sophisticated statistical techniques and machine learning algorithms, researchers can efficiently detect patterns in data relevant to neuroplasticity. This enhanced efficiency can expedite the research process and facilitate the rapid translation of findings into clinical practice (Bohr and Memarzadeh, 2020; Table 2).

### 8.3 AI chatbots and language models for cognitive for patients with neurodegenerative disorders

Chatbots are being used more and more in different areas, such as clinical and medical environments, to assist with therapeutic interventions (Dergaa et al., 2023a). Although they have the potential to provide advantages, it is essential to acknowledge their limits, especially in the context of clinical reasoning and decision-making (Dergaa et al., 2024b; Dergaa et al., 2023b; Dergaa et al., 2024c; Washif et al., 2024). It is important to acknowledge that while AI chatbots and language models offer potential benefits in cognitive rehabilitation, they should be viewed as complementary tools, or co-intelligence, rather than replacements for human expertise. The integration of AI in healthcare requires careful consideration of ethical implications and potential limitations (Bekbolatova et al., 2024).

In early-stage Alzheimer’s disease, AI chatbots provide structured cognitive exercises integrated with physical activity prompts, showing a 40% improvement in exercise adherence compared to standard care (Dergaa et al., 2024c). For Parkinson’s disease patients, chatbot-guided dual-task training combining cognitive challenges with motor exercises has demonstrated enhanced outcomes in both domains (Washif et al., 2024), helping them manage anxiety or sadness. Additionally, chatbots can offer personalized exercise prescriptions to address specific cognitive impairments (Dergaa et al., 2024c, Washif et al., 2024). Through the examination of specific patient profiles, AI tools could propose suitable physical exercises that are by a person’s cognitive capacities and requirements (Dergaa et al., 2024a; Dergaa et al., 2023b).

While chatbots are unable to substitute the knowledge and skills of experienced professionals, they can provide valuable assistance that enhances current treatment strategies (Dergaa et al., 2024a; Dergaa et al., 2024b; Dergaa et al., 2023b; Dergaa et al., 2024c; Washif et al., 2024). This integration can enhance health outcomes for those grappling with the difficulties of neurodegenerative illnesses (Table 2).

### 8.4 Combined AI and physical activity interventions

Initiatives have demonstrated the potential of integrating AI-based interventions with PA programs to address cognitive impairment in neurodegenerative disorders (Khalid et al., 2024). A 12-week randomized controlled trial in Alzheimer’s disease patients (*n* = 120) combined AI-guided exercise prescription with cognitive training, resulting in significantly slower cognitive decline (23% reduction) compared to standard exercise programs (Campbell et al., 2023). In Parkinson’s disease, virtual reality-based exercise systems using AI for real-time movement analysis improved motor function by 35% compared to conventional physiotherapy (Ramalho et al., 2024). For instance, a pilot study combined AI-powered cognitive training with organized PAs for older individuals at risk of dementia, offering personalized interventions based on individual capabilities. The training yielded substantial enhancements in both physical fitness and cognitive performance (Desjardins-Crepeau et al., 2016). A significant number of participants expressed increased motivation and involvement, suggesting that the integration of PA and cognitive training can augment the overall efficacy of the treatment. Another study emphasized the significance of personalization and continuous support to sustain patient involvement (Hickmann et al., 2022).

Additional research supports these conclusions. An example is a program that utilized virtual reality and AI to encourage PA in elderly individuals. This program showed that participants not only enhanced their physical well-being but also gained cognitive advantages (Ramalho et al., 2024). Studies have demonstrated that using AI technologies with fitness routines might enhance compliance and yield better results for persons experiencing cognitive decline (Leung et al., 2022).

In addition, a research investigation examining the amalgamation of AI-powered cognitive training and PA in adults with mild cognitive impairment discovered that this combined strategy led to improved cognitive performance in comparison to each intervention separately (Campbell et al., 2023; Table 2).

### 8.5 Internet of things (IoT)

The applications discussed in the previous subsections of “Integration of AI, machine learning, and language models in cognitive rehabilitation” have the potential to become fully automated and interconnected through the integration of the IoT. The latter refers to the network of physical devices, sensors, and everyday objects embedded with electronics, software, and connectivity, enabling data exchange and communication (Al-Kahtani et al., 2022). In the context of personalized exercise interventions for neurodegenerative disorders, IoT technologies can facilitate the seamless collection and transmission of real-time data from wearable devices, smart home sensors, and other connected gadgets. This data can incorporate various parameters, such as PA levels, sleep patterns, and vital signs, providing a comprehensive view of the patient’s health and response to prescribed exercises. While the integration of IoT with AI, machine learning, and language models for exercise prescription is an emerging area of research, the potential benefits are hugely promising.

An envisioned application involves the continuous monitoring of patients’ PA patterns and vital signs through wearable devices. Machine learning models would automatically analyze the data collected from these IoT devices. Based on the analysis, the models could recommend adjustments to the prescribed exercise program. These recommendations would be communicated to the patient through a conversational AI assistant powered by a large language model. The large language model-based AI assistant would provide personalized guidance, motivation, and feedback, thereby fostering better adherence and engagement with the tailored exercise regimen. While the full realization of this integrated system is still a topic of ongoing research and development, its potential to revolutionize personalized exercise interventions for neurodegenerative disorders is undeniable.

## 9 Conclusion

Incorporating PA into rehabilitation protocols presents a promising avenue for enhancing neuroplasticity and cognitive function in individuals with neurodegenerative disorders. The evidence reviewed suggests that regular PA can positively modify neurogenesis, synaptic plasticity, and neuronal proliferation, leading to significant improvements in memory and executive function. Engaging in regular PA enhances the brain’s ability to adapt and triggers the production of crucial neurotrophic factors. This empowers patients to actively engage in their rehabilitation and promotes overall physical and mental well-being. The interdependence between movement and cognitive processes, as conceptualized by various authors, highlights the profound impact of motor activity on brain function and structure.

In this review, we emphasized the importance of integrating non-pharmacological personalized PA regimens into cognitive rehabilitation protocols. The integration of PA along with cognitive training, potentially augmented by AI technologies, has promising results in enhancing both physical fitness and cognitive performance. Healthcare practitioners should tailor these programs to correspond with the individual requirements and capabilities of patients, guaranteeing involvement and efficacy of outcomes. Future research should focus on elucidating the optimal timing, duration, sequence, and type of cognitive engagement that interacts positively with exercise to promote cognitive and brain health. Furthermore, continuous training for professionals is needed to effectively apply these treatments. In this regard, while AI solutions offer potential benefits in personalizing exercise routines and monitoring progress, it is crucial to maintain a primary emphasis on the intrinsic advantages of PA. The development of AI in healthcare requires careful consideration of ethical implications and potential limitations. Future studies focusing on tangible applications of PA interventions may significantly enhance our ability to manage and mitigate cognitive decline. Exploring modalities that boost cognitive function across diverse patient populations could help individuals regain their standard of living and perform daily tasks more efficiently. Incorporating larger sample sizes would enable investigations to delve into how multiple genes and associated proteins moderate the impact of PA on cognition and brain function. Ultimately, combining PA, cognitive rehabilitation, and emerging technologies presents a multifaceted approach to addressing neurodegenerative disorders in a non-pharmacological way. Synergistically utilizing these elements could improve the quality of life for those affected by cognitive impairment and facilitate prevention and more personalized, effective interventions in neurodegenerative disease management.
